# Cohort profile: Guangdong Pharmaceutical University Occupational Health Cohort in Xishan Coal Electricity Group (GDPU-OHC)

**DOI:** 10.3389/fpubh.2026.1877648

**Published:** 2026-07-27

**Authors:** Haiqing Lin, Huifang Zhang, Zeyuan Zhang, Shiting Yi, Lvrong Li, Yingshi Dai, Yanhui Liu, Hongxia Zhao, Linhui Pang, Liuquan Jiang, Gaisheng Liu, Xiaohua Ye, Xi Fu, Jie Tang, Jisheng Nie, Yingjun Chen

**Affiliations:** 1 The First Affiliated Hospital, Guangdong Pharmaceutical University, Guangzhou, China; 2 Department of Epidemiology and Biostatistics, School of Public Health, Guangdong Pharmaceutical University, Guangzhou, China; 3 Department of Occupational and Environmental Health, School of Public Health, Shanxi Medical University, Taiyuan, China; 4 School of Public Health, Guangdong Pharmaceutical University, Guangzhou, China; 5 Xishan Coal Electricity Corporation Occupational Disease Prevention and Control Institute, Taiyuan, China

**Keywords:** China, coal miners, cohort studies, non-communicable diseases, occupational health

## Abstract

**Purpose:**

Non-communicable diseases (NCDs) are an important public health concern among coal miners, who may experience occupational hazards, demanding working conditions, and unhealthy lifestyles. The Guangdong Pharmaceutical University Occupational Health Cohort in Xishan Coal Electricity Group (GDPU-OHC) is an ambispective occupational cohort established to characterize NCD burden, identify high-risk occupational groups, support future longitudinal analyses, and inform occupational health management in China.

**Participants:**

GDPU-OHC was conducted in Shanxi Province, China, with a retrospective component covering 2009–2022 and a prospective component initiated in May 2023. Among 36,577 workers with routine health examination records in 2023, 25,597 eligible employees (median age: 41.00 years, IQR: 35.00–50.00; 89.19% male) were recruited into the prospective cohort. All baseline participants were asked to provide fasting blood and urine samples. The main cohort is scheduled for follow-up every 3–5 years. Planned sub-studies include occupational protection improvement (*N* = 200), work pattern optimization (*N* = 600), dietary optimization (*N* = 600), and a gut microbiota modulation pilot study (*N* = 50).

**Findings to date:**

Among baseline participants, the crude prevalence of obesity, hypertension, type 2 diabetes, hyperuricemia, and hyperlipidemia was 22.64, 23.18, 13.89, 27.87, and 47.93%, respectively. Occupational hazards, including dust, noise, toxic gasses, and shift work, and lifestyle factors, including high oil/salt intake, smoking, and alcohol consumption, were common in this cohort and will serve as key exposures for future longitudinal analyses of NCD risks. These findings are descriptive; temporal trends and exposure-outcome associations were not assessed in the present cohort profile.

**Future plans:**

The GDPU-OHC will continue prospective follow-up to capture long-term NCD outcomes and repeated occupational health information. Future studies will evaluate longitudinal associations between occupational/lifestyle exposures and NCD risks, develop planned sub-studies, and conduct mechanistic analyses involving oxidative stress, inflammatory markers, metabolomics, and gene–environment interactions. Study findings will be disseminated through serial publications.

## Introduction

In China, occupational populations may be exposed to both workplace hazards and environmental pollutants, which may contribute to the substantial burden of non-communicable diseases (NCDs) and represent an important public health concern (1–3). Coal miners are a particularly relevant occupational group because they may experience chronic exposure to coal dust, high temperatures, noise, hypoxia, and environmental pollution in mining areas, together with long employment duration, high work intensity, and psychological stress. Inadequate health management and safety education may further aggravate health risks in this population. Previous studies have linked coal-mining-related occupational and environmental exposures with sleep disorder, hypertension (4), cardiovascular diseases (CVDs) (5), chronic kidney disease (6–8), dyslipidemia (9), metabolic abnormality (10), reproductive system disease (10), anxiety, depression, and tumors (10, 11). Therefore, targeted occupational health management and safety education for coal miners are important for NCD prevention and control and likely to yield substantial health benefits.

Current NCD management models focus predominantly on community settings, older adults, and legally defined occupational diseases, with comparatively less attention to NCDs in large occupational groups. Evidence from previous studies suggests that occupational personal protection is relevant to workers’ health. For example, studies have reported associations between dust exposure and chronic disease risk, supporting the need for improved occupational safety measures and personal protective equipment (PPE) (12–14). Prior studies have also suggested that interventions addressing workplace and lifestyle factors may improve physical and mental health outcomes (10). For example, a longitudinal study of workers with chronic diseases in the Netherlands from 2010 to 2016 reported that optimizing working patterns was associated with improved health-related employment outcomes (RR = 0.46) (15). A three-week community-based dietary intervention among women in Canada reduced body weight (1.4%), total cholesterol (TC) (14.1%), low-density lipoprotein cholesterol (LDL-C) (16.8%), fasting plasma glucose (FPG) (6.3%), and C-reactive protein (CRP) (14.0%), accompanied by gut microbiome modulation and beneficial microbial metabolites (16). A 12-month dietary intervention in 612 older adults from several European countries showed that a Mediterranean diet increased beneficial microflora such as *Faecalibacterium prausnitzii* and *Roseburia*, while decreasing *Ruminococcus torques* and *Collinsella aerofaciens*, which are associated with frailty (17). In addition, the gut microbiota plays a central role in host homeostasis and metabolic regulation, and probiotics may exert protective effects by preserving microbial balance (18).

Against this background, we established the Guangdong Pharmaceutical University Occupational Health Cohort in Xishan Coal Electricity Group (GDPU-OHC), an ambispective occupational cohort of coal mine workers in Shanxi Province, China. This cohort profile describes the cohort design, baseline characteristics, occupational exposure information, biospecimen collection, and planned follow-up framework. GDPU-OHC is intended to characterize the NCD burden in this occupational population, support future longitudinal analyses of occupational and lifestyle exposures, and provide a platform for planned nested sub-studies, including occupational protection improvement, work pattern optimization, dietary optimization, and gut microbiota modulation.

## Methods and analysis

### Subjects recruited and basic information

**Figure 1:**
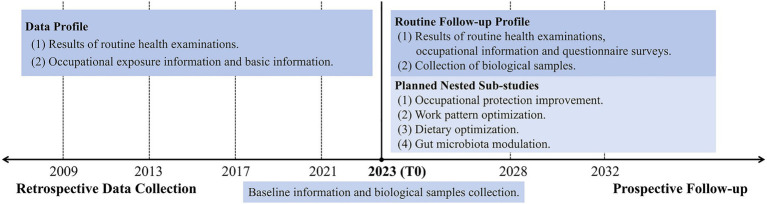
Timeline of data collection, baseline recruitment, and planned follow-up in the GDPU-OHC. (i) Retrospective Data Collection: routine occupational health examination records from 2009 to 2022 were extracted from the electronic health examination system, comprising 529,040 person-examination records. Occupational exposure information and basic demographic information were also collected where available. (ii) T0 (baseline): in 2023, 36,577 workers had routine health examination records and served as the source population for baseline recruitment. Among them, 25,597 eligible participants completed electronic informed consent, the baseline questionnaire, physical examination, and biospecimen collection, and were enrolled in the prospective cohort. (iii) Prospective Follow-up: participants will be followed through periodic cohort follow-up, routine health examinations, occupational information updates, questionnaire surveys, and biospecimen collection where applicable. Planned nested sub-studies include occupational protection improvement, work pattern optimization, dietary optimization, and gut microbiota modulation.

GDPU-OHC is an ambispective cohort study conducted in Shanxi Province, China. From May 2023, 36,577 workers with routine health examination records served as the source population for baseline recruitment. After excluding individuals who could not be contacted, refused the survey, or were unable to participate due to health-related reasons, 36,517 individuals provided electronic informed consent and initiated the baseline survey. After further quality-control exclusions, 25,597 eligible participants were enrolled into the prospective baseline cohort after completing the baseline questionnaire, physical examination, and biospecimen collection. These participants will be followed prospectively every 3–5 years, while selected participants may be invited to participate in planned intervention or pre-post studies. In parallel with the baseline recruitment, historical occupational health examination data from 2009 to 2022 were retrospectively collected to provide pre-baseline health information. The timeline of the GDPU-OHC is depicted in Figure 1. Baseline recruitment and biospecimen collection began after initial ethics approval had been obtained in 2023 [Guangdong Pharmaceutical University Public Health Medical Ethics (2023) No. 5], and a subsequent protocol-specific ethics approval was obtained in 2024 from the Ethics Committee of the First Affiliated Hospital of Guangdong Pharmaceutical University [Medical Ethics Audit (2024) KT No. 98] for follow-up and biospecimen-based analyses. Electronic informed consent was obtained from all participants before questionnaire completion.

The cohort covers more than 10 mining-related job types. Water pumpers, gas inspectors, and electricians/maintenance workers are mainly exposed to noise; support workers, hydraulic support operators, belt operators, locomotive drivers, flotation workers, and heavy media sorters are exposed to coal dust and noise; and coal miners and ventilation workers may be exposed to toxic gasses in addition to coal dust and noise. In contrast, management and service workers are not considered routinely exposed to the monitored occupational hazards.

### Inclusion and exclusion criteria

**Figure 2:**
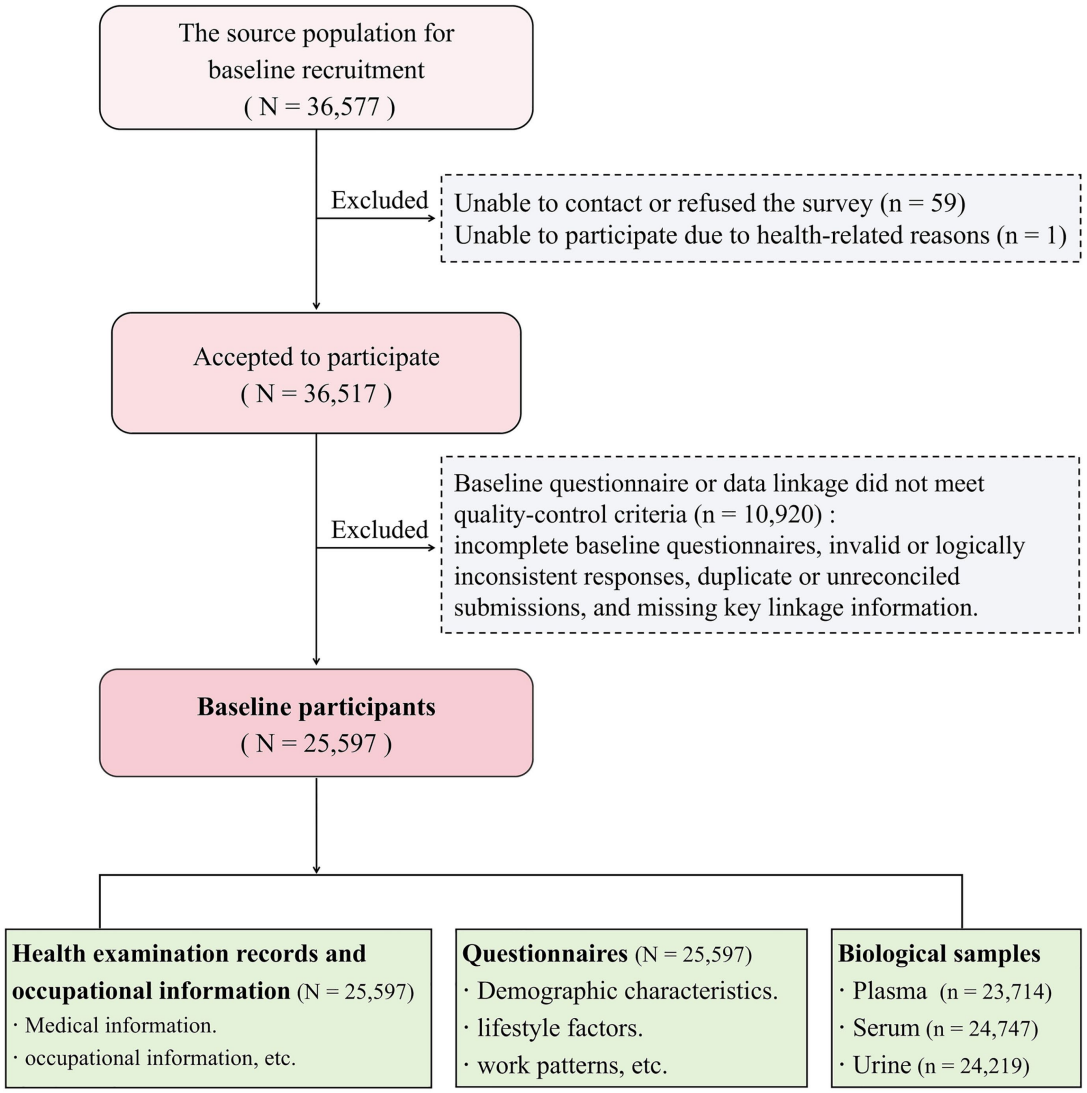
Flow diagram of baseline recruitment and data collection in GDPU-OHC. (i) In 2023, 36,577 workers with routine health examination records served as the source population for baseline recruitment. After excluding individuals who could not be contacted, refused the survey, were unable to participate due to health-related reasons, or whose baseline questionnaire or data linkage did not meet quality-control criteria, 25,597 participants were included in the prospective baseline cohort. (ii) Quality-control exclusions included incomplete baseline questionnaires, invalid or logically inconsistent responses, duplicate or unreconciled submissions, and missing key linkage information. Baseline data included health examination records, questionnaire information, occupational information where available, and collected biospecimens.

The inclusion criteria were: (i) age ≥ 18 years; (ii) ≥ 6 months of employment in the company; (iii) completion of ≥3 physical examinations at cooperating medical institutions during 2009–2022; and (iv) electronic informed consent, completion of the baseline questionnaire and physical examination. The exclusion criteria were: (i) unable to provide an accurate national identification card or key linkage information; (ii) severe physical or mental illness precluding completion of the examination; and (iii) refusal to provide blood or urine samples or to participate in follow-up. The baseline recruitment process is shown in Figure 2.

### Baseline characteristics (2023)

Baseline characteristics are summarized in Table 1. Of the 25,597 baseline participants, 22,829 were male (89.19%) and 2,768 were female (10.81%). The median age was 41.00 years (IQR: 35.00–50.00). The median body weight was 73.00 kg, and the mean height was 170.31 cm. Mining/smelting workers and management and service workers accounted for 83.70 and 10.18%, respectively, and 6.13% had missing occupational-category information. Excessive daily intake of cooking oil and salt was reported by 7.74 and 44.99% of participants, respectively. In total, 25.35% consumed ≥ 12 food types/day and 28.82% consumed ≥ 25 food types/week. Self-reported occupational factors included dust exposure (53.55%), noise exposure (50.80%), toxic gas exposure (28.29%), and shift work (53.47%).

**Table 1:** The baseline characteristics of participants in the GDPU-OHC study.

Variables	Total	Male	Female	*P* ^d^
*N*, *n* (%)	25,597 (100.00)	22,829 (89.19)	2,768 (10.81)	
Age (year)	41.00 (35.00, 50.00)	41.00 (35.00, 50.00)	40.00 (37.00, 46.00)	0.019
Height (cm)^a^	170.31 ± 6.90	171.45 ± 6.16	160.85 ± 5.16	<0.001
Weight (kg)^a^	73.00 (65.00, 82.00)	75.00 (67.00, 83.00)	61.00 (55.00, 67.00)	<0.001
Education, *n* (%)				<0.001
Primary below	303 (1.18)	298 (1.31)	5 (0.18)	
Middle school	3,303 (12.90)	3,154 (13.82)	149 (5.38)	
High school	6,635 (25.92)	6,384 (27.96)	251 (9.07)	
Junior college	11,128 (43.47)	9,082 (39.78)	2046 (73.92)	
College and above	129 (0.50)	107 (0.47)	22 (0.79)	
Missing	4,099 (16.01)	3,804 (16.66)	295 (10.66)	
Ethnicity, *n* (%)				<0.001
Han	21,356 (83.43)	18,913 (82.85)	2,443 (88.26)	
Others	142 (0.55)	112 (0.49)	30 (1.08)	
Missing	4,099 (16.01)	3,804 (16.66)	295 (10.66)	
Night shift, *n* (%)				<0.001
Yes	13,687 (53.47)	13,080 (57.29)	607 (21.92)	
No	10,342 (40.40)	8,530 (37.36)	1812 (65.46)	
Missing	1,568 (6.13)	1,219 (5.34)	349 (12.61)	
Cigarette smoking, *n* (%)				<0.001
Non-smoker	9,416 (36.79)	6,969 (30.53)	2,447 (88.40)	
Current smoker^b^	10,844 (42.36)	10,819 (47.39)	25 (0.90)	
Former smoker	1,238 (4.84)	1,237 (5.42)	1 (0.04)	
Missing	4,099 (16.01)	3,804 (16.66)	295 (10.66)	
Alcohol drinking, *n* (%)				<0.001
Non-drinker	10,300 (40.24)	8,063 (35.32)	2,237 (80.82)	
Current drinker^c^	12,853 (50.21)	12,528 (54.88)	325 (11.74)	
Former drinker	1,162 (4.54)	1,145 (5.02)	17 (0.61)	
Missing	1,282 (5.01)	1,093 (4.79)	189 (6.83)	
BMI, *n* (%)				<0.001
Normal (<24)	8,829 (34.49)	7,276 (31.87)	1,553 (56.11)	
Overweight (24 ≤ BMI < 28)	10,957 (42.81)	10,066 (44.09)	891 (32.19)	
Obesity (≥ 28)	5,790 (22.62)	5,469 (23.96)	321 (11.60)	
Missing	21 (0.08)	18 (0.08)	3 (0.11)	
Diet, *n* (%)				
Oil intake > 30 g/day	1982 (7.74)	1,653 (7.24)	329 (11.89)	<0.001
Oil intake ≤ 30 g/day	22,047 (86.13)	19,957 (87.42)	2090 (75.51)	
Salt intake ≥ 6 g/day	11,515 (44.99)	10,519 (46.08)	996 (35.98)	<0.001
Salt intake < 6 g/day	12,514 (48.89)	11,091 (48.58)	1,423 (51.41)	
≥12 food types/day	6,490 (25.35)	5,761 (25.24)	729 (26.34)	<0.001
≥25 food types/week	7,378 (28.82)	6,588 (28.86)	790 (28.54)	0.030
Missing	1,568 (6.13)	1,219 (5.34)	349 (12.61)	
Occupation, *n* (%)				<0.001
Management and service workers	2,605 (10.18)	1811 (7.93)	794 (28.68)	
Mining/smelting workers	21,424 (83.70)	19,799 (86.73)	1,625 (58.71)	
Missing	1,568 (6.13)	1,219 (5.34)	349 (12.61)	
Exposure, *n* (%)				
Dust	13,707 (53.55)	13,382 (58.62)	325 (11.74)	<0.001
Noise	13,002 (50.80)	12,571 (55.07)	431 (15.57)	<0.001
Toxic gasses	7,241 (28.29)	7,135 (31.25)	106 (3.83)	<0.001
Missing	1,568 (6.13)	1,219 (5.34)	349 (12.61)	
Job category, *n* (%)				<0.001
Support worker	3,821 (14.93)	3,820 (16.73)	1 (0.04)	
Ventilation worker and dust surveyor	692 (2.70)	691 (3.03)	1 (0.04)	
Gas inspector	564 (2.20)	563 (2.47)	1 (0.04)	
Roadheader driver	814 (3.18)	814 (3.57)	0 (0.00)	
Coal digger	1,321 (5.16)	1,321 (5.79)	0 (0.00)	
Hydraulic support operator	266 (1.04)	266 (1.17)	0 (0.00)	
Belt operator	1,193 (4.66)	1,152 (5.05)	41 (1.48)	
Safety inspector	1,326 (5.18)	1,321 (5.79)	5 (0.18)	
Locomotive driver	1,658 (6.48)	1,629 (7.14)	29 (1.05)	
Flotation worker	323 (1.26)	313 (1.37)	10 (0.36)	
Heavy media sorter	197 (0.77)	168 (0.74)	29 (1.05)	
Electrician/maintenance worker	1,608 (6.28)	1,595 (6.99)	13 (0.47)	
Water pumper	491 (1.92)	434 (1.90)	57 (2.06)	
Others	1858 (7.26)	1779 (7.79)	79 (2.85)	
No exposure to occupational hazards	6,770 (26.45)	4,862 (21.30)	1908 (68.93)	
Missing	2,695 (10.53)	2,101 (9.20)	594 (21.46)	

^a^Missing anthropometric data included 21 individuals without height and weight information. ^b^Current smokers were defined as those who smoked at least one cigarette per day in the past 6 months. ^c^Current drinkers were those who drank hard liquor, beer or wine at least once per week during the past 6 months. ^d^Between-group comparisons were performed using *t*-test for normally distributed continuous variables, Wilcoxon rank-sum test for non-normally distributed continuous variables, and chi-square test for categorical variables, as appropriate. *P*-values (*P*) < 0.05 were statistically significant. All *p*-values were two-sided.

### Collaborative framework and cohort organization

GDPU-OHC was initiated by Guangdong Pharmaceutical University in collaboration with Shanxi Medical University and the Xishan Coal Electricity Corporation Occupational Disease Prevention and Control Institute. All three institutions participated in cohort design, participant recruitment, data collection, and follow-up. Guangdong Pharmaceutical University served as the leading institution and was primarily responsible for the overall study design, field recruitment, on-site questionnaire investigation, physical examinations, biospecimen collection and management, occupational exposure assessment, follow-up implementation, data management, and quality control. Shanxi Medical University provided support for field coordination and questionnaire administration, and will further assist with future follow-up activities. The Xishan Coal Electricity Corporation Occupational Disease Prevention and Control Institute provided access to the study sites and de-identified occupational health and medical examination records. Details are provided in Supplementary Table S1.

### Follow-up visits

#### Main cohort

Participants in the main cohort will undergo on-site follow-up every 3–5 years (19). Each follow-up will include questionnaires, physical examinations, collection of biological samples, and laboratory testing, with physiological and biochemical indicators aligned with baseline assessments.

#### Planned nested sub-studies

Selected participants with specific NCDs and comparison participants from the cohort may be invited to participate in planned nested sub-studies. These sub-studies are intended to evaluate feasibility, adherence, changes in clinical indicators, and potential intervention-related outcomes. Additional follow-up will be conducted through telephone interviews, periodic review of medical records, and chronic disease surveillance systems where available. At the time of this cohort profile, these nested sub-studies have not yet been fully implemented or registered. Detailed protocols, additional ethical approval where required, and trial registration will be completed before participant recruitment.

Three protocol-based intervention sub-studies and one quasi-experimental pre-post occupational protection improvement program are planned within GDPU-OHC: (i) occupational protection improvement; (ii) work pattern optimization; (iii) dietary optimization; and (iv) gut microbiota modulation. Before implementation, detailed protocols, additional ethical approval where required, and trial registration will be completed for protocol-based intervention sub-studies. The occupational protection improvement component will be conducted as a quasi-experimental pre-post program rather than a randomized controlled trial.

##### Occupational protection improvement

The PPE for the head, hearing, eyes, respiratory tract, hands, feet, and body will be provided to 200 miners who are frequently exposed to coal dust, noise, and toxic gas, such as support workers, coal miners, and roadheader drivers (20). Miners who previously did not routinely use protective equipment will be required to use safety helmets, dust masks, noise-proof earplugs, gloves, shoe covers, and goggles during underground work. In this pre-post program, PPE use, implementation fidelity, and related occupational protection indicators will be assessed every 3 months for 3 years.

##### Work pattern optimization

A protocol-based intervention sub-study is planned among workers with a specific NCD (N = 600), with random allocation to an intervention group (*N* = 300) and a control group (*N* = 300) (21). The control group maintains the original work patterns and duration. The intervention group will adopt a forward-rotating shift schedule with reduced work duration. Day-shift work will include morning shifts from 8:00 a.m. to 12:00 p.m. and afternoon shifts from 2:00 p.m. to 6:00 p.m.; night-shift work will run from 8:00 p.m. on day 1 to 6:00 a.m. on day 2 (21, 22). Participants in the intervention group will also be encouraged to increase physical activity by 2.5 h per week, and weekly working hours will be reduced from 39 to 30 h (23).

##### Dietary optimization

A canteen-based dietary optimization sub-study is planned among workers with specific NCDs (*N* = 600), including underground and surface workers (24). Staff canteens in each mine will be randomly assigned to an intervention or usual-diet group. The dietary intervention group will include “protein at every meal” (one egg plus one portion of lean meat or tofu); increasing mixed grains to 20%; adjusting the whole-wheat flour ratio from 1:4 to 1:3; providing two vegetables and one portion of fruit per day; and reducing deep-fried dishes by 50%. Each stove will be equipped with a 2-g salt-control spoon and a 10-g oil-restriction jug. According to the dietary intervention protocol, no more than 10 g of salt and 20 g of cooking oil should be used per dish prepared for approximately 50 servings (25). The usual-diet group will maintain the original dietary pattern. All diets contain <300 mg/day cholesterol and include all major food groups with no total calorie restriction to avoid outliers and control fat intake (24).

##### Gut microbiota modulation

Based on prior evidence that short-term probiotic consumption may improve glycemic control, plasma lipids, and inflammatory biomarkers in patients with type 2 diabetes (26–28), a pilot randomized, double-blind, placebo-controlled sub-study is planned to evaluate feasibility, adherence, safety, and exploratory biomarker changes. The planned sample size is 50 participants, with 1:1 allocation to probiotic or placebo groups (26, 29). Participants will receive either Bio-K 50B at 100 billion CFU/day (two capsules/day) or placebo for 12 weeks. Each enteric-coated capsule contains ≥50 billion CFU of synergistic lactobacilli, including *Lactobacillus acidophilus*, *Lacticaseibacillus casei*, and *Lacticaseibacillus rhamnosus* (30).

### Measurement indicators

#### Overview

In 2023, the study compiled retrospective health examination records and occupational exposure information and conducted a new baseline assessment. Biospecimens were collected on site, including urine in 50-mL centrifuge tubes and fasting venous blood in 5-mL tubes, followed by laboratory processing. A structured questionnaire collected information on demographic characteristics, lifestyle, personal and offspring health status, dietary habits, occupational factors, and reproductive history among women (Table 2).

**Table 2:** Summary of questionnaire data collected in the GDPU-OHC project.

Content category	Specific items
Demographic Characteristics	Personal basic information, marital status, educational level, economic income, etc.
Personal Lifestyle Behavior Factors	Smoking, alcohol consumption, sleep and daily routine, dietary habits, frequency of tea/coffee/vinegar/chili consumption, physical activity, electronic device use, etc.
Past Medical History	Previous medical history (diagnosis time, regular medication treatment; including inquiries about musculoskeletal diseases), family history.
Reproductive History (Female)	Age at menarche, pregnancy history, reproductive history, breastfeeding history, contraceptive use history, etc.
Health Status	Skin reactions, allergies, respiratory symptoms, Chronic Obstructive Pulmonary Disease (COPD) symptoms.
Occupational History	Department, job type, physical labor intensity, shift schedule, exposure to occupational hazards, protective equipment use, past work history, etc.
Occupational Stress	Job Demand-Control (JDC) Scale, Copenhagen Burnout Inventory (CBI).

#### Clinical records and biospecimens

From 2009 to 2023, a total of 565,617 occupational and health examination person-records were extracted from the electronic medical examination system. Detailed diagnostic criteria and data sources for each NCD are provided in Supplementary Table S2. During the 2023 baseline occupational health examinations, fasting venous blood and random urine samples were collected by trained medical staff following standardized operating procedures. Blood samples were collected into ethylenediaminetetraacetic acid (EDTA) anticoagulant tubes and serum-separation tubes. Subsequently, samples were transported under temperature-controlled conditions to the laboratory, processed within the same day, and aliquoted to avoid repeated freeze–thaw cycles. Plasma, serum, and urine aliquots were labeled with unique barcode identifiers linked to participant information in the cohort database. Ultimately, 23,714 fasting plasma aliquots (2 mL each), 24,747 serum aliquots (2 mL each), and 24,219 random-urine aliquots (20 mL each) were archived at −80 °C in the GDPU-OHC biobank. All biospecimens were managed using a standardized inventory system. The medical examination and blood biochemistry variables are summarized in Table 3.

**Table 3:** Summary of physical examination data in the GDPU-OHC project.

Content category	Specific items
General Examination	Height, weight, three circumferences (chest circumference, waist circumference, hip circumference), body fat percentage, blood pressure, pulse rate.
Internal Medicine, Surgery, Ophthalmology and Otorhinolaryngology Examination	Cardiac, pulmonary, hepatic, splenic, skin, mucosal, hearing, and gynecological examinations.
Blood Biochemistry Analysis	Red Blood Cells (RBC), Hemoglobin (HGB), Hematocrit (HCT), Mean Corpuscular Volume (MCV), Mean Corpuscular Hemoglobin (MCH), Mean Corpuscular Hemoglobin Concentration (MCHC), Red Blood Cell Distribution Width-Coefficient of Variation (RDW-CV), Platelets (PLT), Mean Platelet Volume (MPV), Platelet Distribution Width (PDW), White Blood Cells (WBC).
Urinalysis	pH, Nitrite (NIT), Glucose (GLU), Specific Gravity (SG), Protein (PRO), Bilirubin (BIL), Urobilinogen (URO), Ketone Bodies (KET), Leukocytes (LEU), Occult Blood (BLD).
Liver Function	Indirect Bilirubin, Total Bilirubin, Direct Bilirubin, Total Serum Protein, Serum Albumin, Alanine Aminotransferase (ALT), Aspartate Aminotransferase (AST), γ-Glutamyl Transpeptidase (GGT), Alkaline Phosphatase (ALP).
Glucose	Fasting Plasma Glucose (FPG), Glycated Hemoglobin (HbA1c).
Renal Function	Blood Urea Nitrogen (BUN), Creatinine (Cr), Uric Acid (UA).
Lipids	Total Cholesterol (TC), Triglycerides (TG), High-Density Lipoprotein Cholesterol (HDL-C), Low-Density Lipoprotein Cholesterol (LDL-C).
Erythrocyte Sedimentation Rate	Erythrocyte Sedimentation Rate (ESR) Measurement.
Cardiovascular Risk Factors	Serum Homocysteine.
Thyroid Function	Anti-Thyroglobulin Antibody, Anti-Thyroid Peroxidase Antibody, Triiodothyronine (T3), Thyroxine (T4), Free Triiodothyronine (FT3), Free Thyroxine (FT4), Thyroid-Stimulating Hormone (TSH).
Tumor Markers	Carbohydrate Antigen 19–9 (CA19-9), Carbohydrate Antigen 125 (CA125), Prostate-Specific Antigen (PSA), Carcinoembryonic Antigen (CEA), Alpha-Fetoprotein (AFP).
Hemorheology	Whole Blood Reduced Viscosity, Erythrocyte Aggregation Index, Erythrocyte Electrophoresis Time, Whole Blood Viscosity, Plasma Viscosity, Erythrocyte Rigidity Index, Erythrocyte Deformation Index, Erythrocyte Internal Viscosity, High/Medium/Low Shear Flow Resistance, Casson Viscosity, Casson Stress, Hemoglobin Concentration, etc.
Helicobacter Pylori Test	Carbon-13/Carbon-14 Urea Breath Test.
Other Examinations	Chest X-ray, Bone Mineral Density (BMD) Test, Lung Function Test, Abdominal/Neck/Prostate/Thyroid Color Doppler Ultrasound, Arteriosclerosis Detection, etc.

#### Occupational and internal exposures

Occupational exposures were characterized using workplace-level monitoring data from annual occupational hazard surveillance reports during 2009–2023, supplemented by job category, work-area information, and the 2023 baseline questionnaire. These reports were generated in accordance with Chinese national occupational health standards and included measurements of coal dust concentrations (mg/m^3^), workplace noise levels (dB(A)), and toxic gas concentrations (mg/m^3^) at relevant occupational posts or work areas. These data were used to describe workplace-level exposure conditions and should not be interpreted as individual-level exposure measurements. Questionnaire-derived information on shift work, PPE use, and other occupational factors will be integrated into future exposure classification and exposure-response analyses. Planned internal-exposure analyses will include phthalate esters, mycotoxins, bisphenol A, pesticides, and per- and polyfluoroalkyl substances. Where feasible, future mechanistic investigations will quantify oxidative stress and inflammatory markers, conduct metabolomics with an emphasis on lipid metabolism pathways, and explore gene–environment interactions and epigenetic regulation.

#### Intervention-relevant biomarkers

In the planned occupational protection improvement and work pattern optimization components, cardiovascular and inflammatory indicators will be monitored, including TC, triglycerides (TG), LDL-C, high-density lipoprotein cholesterol (HDL-C), and CRP. In the planned dietary optimization and gut microbiota modulation sub-studies, biomarkers will focus on chronic metabolic diseases, including FPG, glycated hemoglobin (HbA1c), insulin, urine microalbumin-to-creatinine ratio, serum creatinine, alanine aminotransferase (ALT), and aspartate aminotransferase (AST). These panels will be repeated at predefined follow-up points in future sub-study protocols.

#### Quality control

Retrospective records from different years were harmonized using a unified data dictionary, and changes in laboratory methods, assays, and reference ranges were documented during data cleaning. Duplicate records were identified using participant identifiers and examination dates, and resolved by retaining the most complete record. Outliers were flagged using clinically plausible ranges and the interquartile range (IQR) method, followed by manual review against source records. Missing data were quantified for each variable. For categorical variables, missing responses were reported as a separate category where applicable, whereas continuous variables were summarized using available non-missing values, with variable-specific denominators reported when necessary. Biospecimens were processed within 4 h after collection, aliquoted, stored at −80 °C, and tracked using barcode-linked records. Two independent analysts checked data completeness and logical consistency using de-identified participant IDs, and all cleaning procedures were documented in an audit trail.

### Statistical analysis

Descriptive statistics were used to summarize the characteristics of the cohort. Normality of continuous variables was assessed using histograms and Q-Q plots. Normally distributed continuous variables were presented as mean ± standard deviation (SD) and compared using independent-samples *t*-tests. Non-normally distributed variables were presented as median with IQR and compared using Wilcoxon rank-sum tests. Categorical variables were expressed as frequencies and percentages and compared using chi-square tests or Fisher’s exact test. Missing data were retained and presented as a separate “missing” category where applicable.

No adjusted regression models, standardized temporal trend analyses, or repeated-measurement longitudinal models were applied in the present cohort profile. Therefore, *p* values should be interpreted as descriptive between-group comparisons rather than evidence of causal or adjusted associations. All analyses were performed using R software version 4.5.2 (R Foundation for Statistical Computing, Vienna, Austria). Two-sided *p*-values < 0.05 were considered statistically significant.

## Findings to date

Table 4 shows that among the 25,597 baseline participants, the median BMI was 25.34 kg/m^2^ (IQR: 23.05–27.70). The crude prevalence of overweight and obesity was 42.84 and 22.64%, respectively. The median systolic and diastolic blood pressure were 126 mmHg (IQR: 115–138) and 80 mmHg (IQR: 72–89), respectively, and the crude prevalence of hypertension was 23.18%. The crude prevalence of type 2 diabetes, hyperuricemia, and hyperlipidemia was 13.89, 27.87, and 47.93%, respectively. The corresponding data among the 36,577 workers with routine health examination records in 2023 are now summarized separately in Supplementary Table S3.

**Table 4:** Physical examination indicators and prevalence of non-communicable diseases in the 2023 baseline population.

Variables and NCDs^a,b,c,d^	Total	Male	Female	*P* ^e^
*N*, *n* (%)	25,597 (100.00)	22,829 (89.19)	2,768 (10.81)	
Common indicators and specific NCDs				
BMI, kg/m^2^	25.34 (23.05, 27.70)	25.53 (23.32, 27.78)	23.44 (21.50, 25.77)	<0.001
Overweight, *n* (%)	10,957 (42.84)	10,066 (44.13)	891 (32.22)	<0.001
Obesity, *n* (%)	5,790 (22.64)	5,469 (23.98)	321 (11.61)	<0.001
Missing, *n* (%)	21 (0.08)	18 (0.08)	3 (0.11)	
SBP, mmHg	126.00 (115.00, 138.00)	127.00 (116.00, 139.00)	120.00 (110.00, 132.00)	<0.001
DBP, mmHg	80.00 (72.00, 89.00)	81.00 (73.00, 89.00)	75.00 (68.00, 83.00)	<0.001
Hypertension, *n* (%)	4,046 (23.18)	3,769 (24.51)	277 (13.32)	<0.001
Missing, *n* (%)	8,140 (31.80)	7,451 (32.64)	689 (24.89)	
WBC (×10^9^/L)	5.89 (4.99, 6.98)	5.95 (5.07, 7.04)	5.35 (4.49, 6.38)	<0.001
RBC (×10^12^/L)	5.29 (5.00, 5.56)	5.34 (5.07, 5.60)	4.74 (4.48, 5.02)	<0.001
PLT (×10^9^/L)	222.00 (189.00, 260.00)	219.00 (186.00, 256.00)	251.00 (214.00, 293.00)	<0.001
HGB (g/L)	156.00 (148.00, 164.00)	158.00 (151.00, 165.00)	136.00 (127.00, 142.00)	<0.001
FPG (mmol/L)	5.10 (4.80, 5.50)	5.10 (4.90, 5.50)	4.90 (4.70, 5.30)	<0.001
HbA1c (%)	5.50 (5.20, 5.80)	5.50 (5.20, 5.80)	5.40 (5.20, 5.70)	<0.001
Type 2 diabetes, *n* (%)	1848 (13.89)	1748 (14.69)	100 (7.09)	<0.001
Missing, *n* (%)	12,289 (48.01)	10,932 (47.89)	1,357 (49.02)	
Uric acid (μmol/L)	364.00 (306.00, 430.00)	374.00 (318.00, 438.00)	280.00 (239.00, 332.00)	<0.001
Hyperuricemia, *n* (%)	7,096 (27.87)	6,962 (30.61)	134 (4.93)	<0.001
Missing, *n* (%)	135 (0.53)	85 (0.37)	50 (1.81)	
TC (mmol/L)	4.41 (3.86, 5.01)	4.40 (3.85, 5.01)	4.48 (3.96, 5.06)	<0.001
TG (mmol/L)	1.47 (0.99, 2.26)	1.52 (1.02, 2.34)	1.11 (0.80, 1.61)	<0.001
HDL-C (mmol/L)	1.08 (0.92, 1.27)	1.06 (0.91, 1.24)	1.27 (1.08, 1.48)	<0.001
LDL-C (mmol/L)	2.58 (2.16, 3.03)	2.58 (2.16, 3.04)	2.55 (2.14, 2.99)	0.014
Hyperlipidemia, *n* (%)	11,854 (47.93)	11,243 (51.02)	611 (22.68)	<0.001
Missing, *n* (%)	867 (3.39)	793 (3.47)	74 (2.67)	
Other indicators and NCDs				
ALT (U/L)	35.00 (30.00, 42.00)	35.00 (31.00, 43.00)	28.00 (26.00, 33.00)	<0.001
AST (U/L)	28.00 (24.00, 34.00)	28.00 (24.00, 34.00)	23.00 (20.00, 27.00)	<0.001
ALP (U/L)	107.00 (98.00, 121.00)	108.00 (101.00, 123.00)	92.00 (87.00, 98.00)	<0.001
GGT (U/L)	43.00 (38.00, 49.00)	45.00 (39.00, 49.00)	38.00 (35.00, 41.00)	<0.001
Total bilirubin (μmol/L)	15.47 (12.63, 20.12)	15.76 (12.72, 20.47)	13.29 (12.27, 16.75)	<0.001
BUN (mmol/L)	5.40 (4.60, 6.40)	5.50 (4.70, 6.50)	4.60 (3.90, 5.50)	<0.001
Serum creatinine (μmol/L)	68.00 (62.00, 75.00)	69.00 (64.00, 76.00)	53.00 (48.00, 59.00)	<0.001
Gallstone, *n* (%)	559 (2.27)	450 (2.05)	109 (4.08)	<0.001
Missing, *n* (%)	932 (3.64)	834 (3.65)	98 (3.54)	
Fatty liver, *n* (%)	7,105 (28.81)	6,660 (30.28)	445 (16.67)	<0.001
Missing, *n* (%)	932 (3.64)	834 (3.65)	98 (3.54)	
Kidney stone, *n* (%)	571 (2.32)	541 (2.46)	30 (1.12)	<0.001
Missing, *n* (%)	932 (3.64)	834 (3.65)	98 (3.54)	
Cirrhosis, n (%)	9 (0.04)	9 (0.04)	0 (0.00)	0.610
Missing, *n* (%)	932 (3.64)	834 (3.65)	98 (3.54)	
Hepatic cyst, *n* (%)	1,508 (6.11)	1,361 (6.19)	147 (5.51)	0.178
Missing, *n* (%)	932 (3.64)	834 (3.65)	98 (3.54)	
COPD, *n* (%)	268 (1.05)	256 (1.12)	12 (0.43)	0.001
Missing, *n* (%)	0 (0.00)	0 (0.00)	0 (0.00)	
CVDs, *n* (%)	421 (1.75)	416 (1.93)	5 (0.21)	<0.001
Missing, *n* (%)	1,568 (6.13)	1,219 (5.34)	349 (12.61)	

^a^NCDs, non-communicable diseases; BMI, body mass index; SBP, systolic blood pressure; DBP, diastolic blood pressure; WBC, white blood cell count; RBC, red blood cell count; PLT, platelet count; HGB, hemoglobin; FPG, fasting plasma glucose; HbA1c, glycated hemoglobin; TC, total cholesterol; TG, triglycerides; HDL-C, high-density lipoprotein cholesterol; LDL-C, low-density lipoprotein cholesterol; ALT, alanine aminotransferase; AST, aspartate aminotransferase; ALP, alkaline phosphatase; GGT, gamma-glutamyl transferase; BUN, blood urea nitrogen; COPD, chronic obstructive pulmonary disease; CVDs, cardiovascular diseases. ^b^The definitions for NCDs are described in Supplementary Table S2. ^c^Prevalence estimates were calculated among participants with available data for the relevant outcome within the 2023 baseline cohort as the denominator, while percentages for missing data were calculated using the full baseline population in 2023 (*N* = 25,597) as the denominator. ^d^Missing data for NCDs are presented in the table. Missing data for continuous variables include BMI (*n* = 21); SBP and DBP (*n* = 8,140); WBC (*n* = 53); RBC (*n* = 55); PLT (*n* = 52); HGB (*n* = 55); FPG (*n* = 894); HbA1c (*n* = 12,729); uric acid (*n* = 135); TC (*n* = 867); TG (*n* = 877); HDL-C (*n* = 866); LDL-C (*n* = 880); ALT (*n* = 2,150); AST (*n* = 2,028); ALP (*n* = 868); GGT (*n* = 745); total bilirubin (*n* = 871); BUN (*n* = 140); serum creatinine (*n* = 139). When biomarker data were partially missing, NCD was classified based on available measurements meeting the diagnostic threshold; participants with insufficient data for reliable classification were treated as missing. ^e^Between-group comparisons were performed using Wilcoxon rank-sum test for non-normally distributed continuous variables, and chi-square test or Fisher’s exact test for categorical variables, as appropriate. *P*-values (*P*) < 0.05 were statistically significant. All *P*-values were two-sided.

Baseline NCD prevalence varied across occupational categories. Among mining/smelting workers, the prevalence of hypertension, type 2 diabetes, hyperuricemia, and hyperlipidemia was 23.24% (3,430/14,762), 13.68% (1,504/10,996), 28.05% (5,974/21,301), and 48.32% (9,944/20,581), respectively. Among management and service workers, the corresponding prevalence was 22.96% (368/1,603), 15.63% (212/1,356), 27.96% (727/2,600), and 46.76% (1,211/2,590), respectively. In selected job groups, support workers had a crude prevalence of 20.34% for hypertension, 10.64% for type 2 diabetes, 26.77% for hyperuricemia, and 47.61% for hyperlipidemia; belt operators had a crude prevalence of 21.86, 15.28, 31.01, and 49.20%, respectively. Detailed occupational-category-specific prevalence and missing data are provided in Supplementary Tables S4, S5.

Occupational and lifestyle factors were common in this cohort, as described in the baseline characteristics. These variables will be used as candidate exposures in future longitudinal analyses. No temporal trend analysis, adjusted occupational risk model, or exposure-response analysis was performed in the present cohort profile.

## Discussion

GDPU-OHC is an ambispective occupational cohort established among coal mine workers from a large coal-electricity group in Shanxi Province. This cohort provides detailed baseline information on occupational characteristics, lifestyle factors, health examination indicators, NCD burden, and biospecimen collection. The cohort may serve as a useful research platform for future longitudinal studies of occupational and lifestyle exposures, NCD progression, and mechanistic biomarkers in similar occupational settings.

Compared with general chronic disease surveillance systems, GDPU-OHC had several occupationally specific features. First, it focuses on a large workforce exposed to coal-mining-related hazards, including dust, noise, toxic gasses, and shift work. Second, it combines baseline questionnaire data, routine health examination records, workplace-level occupational hazard monitoring data, and biospecimens. Third, it provides a framework for planned nested sub-studies, including work pattern optimization, dietary optimization, gut microbiota modulation, and a quasi-experimental pre-post occupational protection improvement program. These components have not yet been fully implemented or registered, and their findings will require separate protocol-based analyses in future studies.

The baseline findings suggest a substantial burden of metabolic and cardiovascular NCDs in this occupational population. Crude NCD prevalence varied across occupational categories, but these differences were unadjusted and may reflect differences in age, sex, job tenure, work tasks, lifestyle factors, medical surveillance participation, and data completeness (31). Therefore, the present findings should be interpreted as descriptive cohort characteristics rather than evidence of causal occupational effects. Future disease-specific studies will use longitudinal models and adjusted analyses to evaluate occupational and lifestyle determinants of NCD risk.

This cohort study has several strengths. First, GDPU-OHC is a large ambispective occupational cohort integrating baseline questionnaires, routine health examination records, occupational information, workplace-level hazard monitoring data, and biospecimens. This multi-source structure provides a valuable platform for characterizing NCD burden and occupational health profiles among coal mine workers. Second, the program includes a prospective follow-up framework, which will enable future longitudinal analyses of NCD incidence, progression, and changes in clinical indicators. Third, it provides a framework for planned nested sub-studies and a quasi-experimental pre-post occupational protection improvement program, which may support future protocol-based research on occupational and lifestyle health-management strategies in this population. Finally, the stored biospecimens enable mechanistic studies of internal exposures, immunologic markers, lipid metabolism, and gene–environment interactions.

However, several limitations should also be noted. First, the present cohort profile is descriptive; no adjusted regression models, standardized temporal trend analyses, exposure-response analyses, or repeated-measurement longitudinal models were performed. Second, large-scale retrospective health examination records may contain missing data or changes in laboratory methods, reference ranges, and health-service providers over time, which may affect data comparability. Third, workplace monitoring reports and job categories were used as workplace-level exposure proxies, and individual-level exposure misclassification may occur (32). Future studies will develop job-exposure matrices and cumulative exposure indices by integrating job history, work area, employment duration, monitoring data, and PPE-use information where available. Fourth, requiring ≥ 3 historical health examinations may introduce healthy worker or survivor bias. Fifth, the planned PPE component lacks individual-level randomization and a concurrent control group, making future PPE-related findings susceptible to time trends and uncontrolled confounding. Finally, because GDPU-OHC was established in a single coal-electricity group in Shanxi Province, the findings may not be fully generalizable to all coal-mining areas or occupational populations in China. Future multi-center studies involving different coal-mining regions and enterprise types are needed to improve external validity.

## Data Availability

The datasets supporting the findings of this study are not publicly available due to privacy, ethical, and institutional restrictions. Further inquiries regarding the data may be directed to the corresponding authors.
