# Supplementary material for: Cohort profile: Guangdong Pharmaceutical University Occupational Health Cohort in Xishan Coal Electricity Group (GDPU-OHC)
Source: Front Public Health. 2026 Jul 27;14:1877648. doi: 10.3389/fpubh.2026.1877648 (PMC13454098; doi:10.3389/fpubh.2026.1877648)
Supplement: Supplementary file 1 [file supplementary_file_1.docx]

**Supplementary Materials**

**Cohort Profile:** **Guangdong Pharmaceutical University Occupational Health Cohort in Xishan Coal Electricity Group (GDPU-OHC)**

Haiqing Lin^1,2†^, Huifang Zhang^3†^, Zeyuan Zhang^1,2^, Shiting Yi^1,2^, Lvrong Li^4^, Yingshi Dai^4^, Yanhui Liu^4^, Hongxia Zhao^5^, Linhui Pang^5^, Liuquan Jiang^5^, Gaisheng Liu^5^, Xiaohua Ye^4^, Xi Fu^4^, Jie Tang^4^, Jisheng Nie^3^* and Yingjun Chen^1,2^*

*^1^The First Affiliated Hospital, Guangdong Pharmaceutical University, Guangzhou, China
^2^Department of Epidemiology and Biostatistics, School of Public Health, Guangdong Pharmaceutical University, Guangzhou, China
^3^Department of Occupational and Environmental Health, School of Public Health, Shanxi Medical University, Taiyuan, China
^4^School of Public Health, Guangdong Pharmaceutical University, Guangzhou, China
^5^Xishan Coal Electricity Corporation Occupational Disease Prevention and Control Institute, Taiyuan, China*

**Table of contents**

[Supplementary Table S1 Roles and responsibilities of collaborating institutions in GDPU-OHC.	1](#_Toc3197)

[Supplementary Table S2 Definition of non-communicable diseases.	2](#_Toc32680)

[Supplementary Table S3 Physical examination indicators and crude prevalence of non-communicable diseases among workers with routine health examination records in 2023 (N = 36,577).	3](#_Toc25072)

[Supplementary Table S4 Crude prevalence of non-communicable diseases by broad occupational category.	5](#_Toc11504)

[Supplementary Table S5 Crude prevalence of non-communicable diseases by detailed occupational group.	6](#_Toc18133)

# **Supplementary Table S1** Roles and responsibilities of collaborating institutions in GDPU-OHC.

| **Institution** | **Roles and responsibilities** |
| --- | --- |
| Guangdong Pharmaceutical University | Leading institution; overall cohort design; field recruitment; on-site questionnaire investigation; physical examinations; biospecimen collection and management; occupational exposure assessment; follow-up implementation; data management; statistical analysis; and quality control. |
| Shanxi Medical University | Providing support for field coordination and questionnaire administration; as well as assistance with future follow-up activities. |
| Xishan Coal Electricity Corporation Occupational Disease Prevention and Control Institute | Provision of study sites; support for access to local occupational health resources; and provision of de-identified occupational health and medical examination records. |

# **Supplementary Table S2** Definition of non-communicable diseases.

| **NCDs** | **Definition ^a, b^** |
| --- | --- |
| Overweight | Overweight was defined as 24 ≤ BMI < 28 kg/m^2^. |
| Obesity | Obesity was defined as BMI ≥ 28 kg/m^2^. |
| Hypertension | Hypertension was defined as SBP ≥ 140 mmHg or DBP ≥ 90 mmHg. |
| Type 2 diabetes | Type 2 diabetes was defined as FPG ≥ 7.0 mmol/L or HbA1c ≥ 6.5%. |
| Hyperlipidemia | Hyperlipidemia was defined as meeting any of the following criteria: TC ≥ 6.2 mmol/L, TG ≥ 2.3 mmol/L, LDL-C ≥ 4.1 mmol/L, HDL-C < 1.0 mmol/L, or based on additional available data as assessed by the investigators. |
| Hyperuricemia | Hyperuricemia was defined as serum uric acid > 420 μmol/L. |
| Gallstone | Gallstone was defined as the presence of stones in the gallbladder on imaging examinations, as diagnosed by a physician. |
| Fatty liver | Fatty liver was defined as the presence of hepatic steatosis on imaging examinations, as diagnosed by a physician. |
| Kidney stone | Kidney stone was defined as the presence of stones in the left kidney, right kidney, or both kidneys on imaging examinations, confirmed by physician diagnosis. |
| Cirrhosis | Cirrhosis was defined as a diagnosis of cirrhosis based on imaging findings and clinical assessment by a physician. |
| Hepatic cyst | Hepatic cyst was defined as the presence of cystic lesions in the liver on imaging examinations, as diagnosed by a physician. |
| COPD | Chronic obstructive pulmonary disease (COPD) was defined as physician‑diagnosed chronic bronchitis and/or emphysema with airflow obstruction. |
| CVDs | Cardiovascular diseases (CVDs) were defined as coronary heart disease, stroke, or other physician-diagnosed cardiovascular conditions. |

**^a^** NCDs, non-communicable diseases; BMI, body mass index; SBP, systolic blood pressure; DBP, diastolic blood pressure; FPG, fasting plasma glucose; HbA1c, glycated hemoglobin; TC, total cholesterol; TG, triglycerides; LDL-C, low-density lipoprotein cholesterol; HDL-C, high-density lipoprotein cholesterol.
**^b^** NCD definitions are provided in this table. Missing values for NCD outcomes defined from one or more clinical measurements may differ from those for individual continuous biomarkers.

# **Supplementary Table S3** Physical examination indicators and crude prevalence of non-communicable diseases among workers with routine health examination records in 2023 (N = 36,577).

| **Variables and NCDs ^a, b, c, d^** | **Overall** |
| --- | --- |
| BMI, kg/m^2^ | 25.28 (22.96, 27.68) |
| Overweight, n (%) | 15305 (42.21) |
| Obesity, n (%) | 8125 (22.41) |
| Missing, n (%) | 314 (0.86) |
| SBP, mmHg | 126.00 (115.00, 138.00) |
| DBP, mmHg | 80.00 (72.00, 89.00) |
| Hypertension, n (%) | 5812 (23.52) |
| Missing, n (%) | 11867 (32.44) |
| WBC (×10^9^/L) | 5.95 (5.04, 7.06) |
| RBC (×10^12^/L) | 5.25 (4.95, 5.53) |
| PLT (×10^9^/L) | 225.00 (191.00, 263.00) |
| HGB (g/L) | 156.00 (147.00, 164.00) |
| FPG (mmol/L) | 5.10 (4.90, 5.50) |
| HbA1c (%) | 5.40 (5.20, 5.80) |
| Type 2 diabetes, n (%) | 2541 (14.22) |
| Missing, n (%) | 18708 (51.15) |
| Uric acid (µmol/L) | 361.00 (301.00, 428.00) |
| Hyperuricemia, n (%) | 9741 (27.05) |
| Missing, n (%) | 565 (1.54) |
| TC (mmol/L) | 4.44 (3.88, 5.06) |
| TG (mmol/L) | 1.46 (0.98, 2.24) |
| HDL-C (mmol/L) | 1.09 (0.93, 1.28) |
| LDL-C (mmol/L) | 2.60 (2.17, 3.07) |
| Hyperlipidemia, n (%) | 15823 (46.56) |
| Missing, n (%) | 2593 (7.09) |
| ALT (U/L) | 35.00 (29.00, 41.00) |
| AST (U/L) | 27.00 (23.00, 33.00) |
| ALP (U/L) | 106.00 (98.00, 121.00) |
| GGT (U/L) | 43.00 (38.00, 49.00) |
| Total bilirubin (µmol/L) | 15.31 (12.67, 19.95) |
| BUN (mmol/L) | 5.40 (4.60, 6.40) |
| Serum creatinine (µmol/L) | 68.00 (62.00, 75.00) |
| Gallstone, n (%) | 796 (2.38) |
| Missing, n (%) | 3143 (8.59) |
| Fatty liver, n (%) | 9643 (28.84) |
| Missing, n (%) | 3143 (8.59) |
| Kidney stone, n (%) | 746 (2.23) |
| Missing, n (%) | 3143 (8.59) |
| Cirrhosis, n (%) | 12 (0.04) |
| Missing, n (%) | 3143 (8.59) |
| Hepatic cyst, n (%) | 2171 (6.49) |
| Missing, n (%) | 3143 (8.59) |
| COPD, n (%) | 268 (1.05) |
| Missing, n (%) | 10980 (30.02) |
| CVDs, n (%) | 421 (1.75) |
| Missing, n (%) | 12548 (34.31) |

**^a^** NCDs, non-communicable diseases; BMI, body mass index; SBP, systolic blood pressure; DBP, diastolic blood pressure; WBC, white blood cell count; RBC, red blood cell count; PLT, platelet count; HGB, hemoglobin; FPG, fasting plasma glucose; HbA1c, glycated hemoglobin; TC, total cholesterol; TG, triglycerides; HDL-C, high-density lipoprotein cholesterol; LDL-C, low-density lipoprotein cholesterol; ALT, alanine aminotransferase; AST, aspartate aminotransferase; ALP, alkaline phosphatase; GGT, gamma-glutamyl transferase; BUN, blood urea nitrogen; COPD, chronic obstructive pulmonary disease; CVDs, cardiovascular diseases.

**^b^** The definitions for NCDs are described in Supplementary Table S2.

**^c^** Prevalence estimates were calculated among workers with available relevant outcome data as the denominator, whereas percentages for missing data were calculated using all workers with routine health examination records in 2023 (N = 36,577) as the denominator.

**^d^** Missing data for NCDs are presented in the table. Missing data for continuous variables include BMI (n = 314); SBP and DBP (n = 11,867); WBC and RBC (n = 371); PLT (n = 369); HGB (n = 372); FPG (n = 2,561); HbA1c (n = 19,316); uric acid (n = 565); TC (n = 2,565); TG (n = 2,575); HDL-C (n = 2,599); LDL-C (n = 2,612); ALT (n = 2,938); AST (n = 2,782); ALP (n = 2,564); GGT (n = 2,406); total bilirubin (n = 2,566); BUN (n = 572); and serum creatinine (n = 571). When biomarker data were partially missing, NCD was classified based on available measurements meeting the diagnostic threshold; participants with insufficient data for reliable classification were treated as missing.

# **Supplementary Table S4** Crude prevalence of non-communicable diseases by broad occupational category.

| **NCDs** ^a, b, c^ | **Mining/smelting workers  n = 21424** | **Management and service workers  n = 2605** | ***P*** ^d^ |
| --- | --- | --- | --- |
| **Overweight** | 9241 (43.17%) | 1082 (41.57%) | 0.124 |
| **Obesity** | 4883 (22.81%) | 599 (23.01%) | 0.837 |
| Missing | 18 (0.08%) | 2 (0.08%) |  |
| **Hypertension** | 3430 (23.24%) | 368 (22.96%) | 0.826 |
| Missing | 6662 (31.10%) | 1002 (38.46%) |  |
| **Type 2 diabetes** | 1504 (13.68%) | 212 (15.63%) | 0.054 |
| Missing | 10428 (48.67%) | 1249 (47.95%) |  |
| **Hyperuricemia** | 5974 (28.05%) | 727 (27.96%) | 0.947 |
| Missing | 123 (0.57%) | 5 (0.19%) |  |
| **Hyperlipidemia** | 9944 (48.32%) | 1211 (46.76%) | 0.140 |
| Missing | 843 (3.93%) | 15 (0.58%) |  |
| **Gallstone** | 449 (2.19%) | 74 (2.88%) | 0.032 |
| Missing | 887 (4.14%) | 32 (1.23%) |  |
| **Fatty liver** | 5875 (28.61%) | 827 (32.14%) | < 0.001 |
| Missing | 887 (4.14%) | 32 (1.23%) |  |
| **Kidney stone** | 486 (2.37%) | 58 (2.25%) | 0.776 |
| Missing | 887 (4.14%) | 32 (1.23%) |  |
| **Cirrhosis** | 8 (0.04%) | 1 (0.04%) | 1.000 |
| Missing | 887 (4.14%) | 32 (1.23%) |  |
| **Hepatic cyst** | 1265 (6.16%) | 138 (5.36%) | 0.121 |
| Missing | 887 (4.14%) | 32 (1.23%) |  |
| **COPD** | 229 (1.07%) | 24 (0.92%) | 0.552 |
| Missing | 0 (0.00%) | 0 (0.00%) |  |
| **CVDs** | 372 (1.74%) | 49 (1.88%) | 0.651 |
| Missing | 0 (0.00%) | 0 (0.00%) |  |

^a^ NCDs, non-communicable diseases; COPD, chronic obstructive pulmonary disease; CVDs, cardiovascular diseases.

^b^ The definitions for NCDs are described in Supplementary Table S2.
**^c^** Prevalence estimates were calculated using participants with available data for the relevant outcome within each occupational category as the denominator, whereas missing-data percentages were calculated using the total number of participants in the corresponding occupational category as the denominator.

^d^ Between-group comparisons were performed using the chi-square test or Fisher's exact test for categorical variables, as appropriate. *P*-values (*P*) < 0.05 were statistically significant. All *p*-values were two-sided.

# **Supplementary Table S5** Crude prevalence of non-communicable diseases by detailed occupational group.

| **Occupation** | **NCDs** ^a, b, c^ | **Total, n** | **Cases, n** | **Missing, n** | **Crude prevalence, %** | **Missing, %** |
| --- | --- | --- | --- | --- | --- | --- |
| **Support worker** | Overweight | 3821 | 1632 | 2 | 42.73 | 0.05 |
|  | Obesity | 3821 | 863 | 2 | 22.60 | 0.05 |
|  | Hypertension | 3821 | 504 | 1343 | 20.34 | 35.15 |
|  | Type 2 diabetes | 3821 | 185 | 2083 | 10.64 | 54.51 |
|  | Hyperuricemia | 3821 | 1022 | 3 | 26.77 | 0.08 |
|  | Hyperlipidemia | 3821 | 1691 | 269 | 47.61 | 7.04 |
|  | Gallstone | 3821 | 47 | 274 | 1.33 | 7.17 |
|  | Fatty liver | 3821 | 916 | 274 | 25.82 | 7.17 |
|  | Kidney stone | 3821 | 77 | 274 | 2.17 | 7.17 |
|  | Cirrhosis | 3821 | 1 | 274 | 0.03 | 7.17 |
|  | Hepatic cyst | 3821 | 182 | 274 | 5.13 | 7.17 |
|  | COPD | 3821 | 38 | 0 | 0.99 | 0.00 |
|  | CVDs | 3821 | 41 | 111 | 1.11 | 2.90 |
| **Ventilation worker and dust surveyor** | Overweight | 692 | 306 | 0 | 44.22 | 0.00 |
|  | Obesity | 692 | 183 | 0 | 26.45 | 0.00 |
|  | Hypertension | 692 | 96 | 233 | 20.92 | 33.67 |
|  | Type 2 diabetes | 692 | 35 | 419 | 12.82 | 60.55 |
|  | Hyperuricemia | 692 | 225 | 0 | 32.51 | 0.00 |
|  | Hyperlipidemia | 692 | 351 | 5 | 51.09 | 0.72 |
|  | Gallstone | 692 | 10 | 5 | 1.46 | 0.72 |
|  | Fatty liver | 692 | 217 | 5 | 31.59 | 0.72 |
|  | Kidney stone | 692 | 18 | 5 | 2.62 | 0.72 |
|  | Cirrhosis | 692 | 0 | 5 | 0.00 | 0.72 |
|  | Hepatic cyst | 692 | 38 | 5 | 5.53 | 0.72 |
|  | COPD | 692 | 6 | 0 | 0.87 | 0.00 |
|  | CVDs | 692 | 7 | 23 | 1.05 | 3.32 |
| **Gas inspector** | Overweight | 564 | 216 | 0 | 38.30 | 0.00 |
|  | Obesity | 564 | 121 | 0 | 21.45 | 0.00 |
|  | Hypertension | 564 | 92 | 83 | 19.13 | 14.72 |
|  | Type 2 diabetes | 564 | 31 | 384 | 17.22 | 68.09 |
|  | Hyperuricemia | 564 | 207 | 0 | 36.70 | 0.00 |
|  | Hyperlipidemia | 564 | 276 | 2 | 49.11 | 0.35 |
|  | Gallstone | 564 | 10 | 2 | 1.78 | 0.35 |
|  | Fatty liver | 564 | 143 | 2 | 25.44 | 0.35 |
|  | Kidney stone | 564 | 10 | 2 | 1.78 | 0.35 |
|  | Cirrhosis | 564 | 0 | 2 | 0.00 | 0.35 |
|  | Hepatic cyst | 564 | 29 | 2 | 5.16 | 0.35 |
|  | COPD | 564 | 7 | 0 | 1.24 | 0.00 |
|  | CVDs | 564 | 6 | 31 | 1.13 | 5.50 |
| **Roadheader driver** | Overweight | 814 | 346 | 0 | 42.51 | 0.00 |
|  | Obesity | 814 | 165 | 0 | 20.27 | 0.00 |
|  | Hypertension | 814 | 151 | 72 | 20.35 | 8.85 |
|  | Type 2 diabetes | 814 | 37 | 338 | 7.77 | 41.52 |
|  | Hyperuricemia | 814 | 195 | 0 | 23.96 | 0.00 |
|  | Hyperlipidemia | 814 | 299 | 15 | 37.42 | 1.84 |
|  | Gallstone | 814 | 12 | 18 | 1.51 | 2.21 |
|  | Fatty liver | 814 | 163 | 18 | 20.48 | 2.21 |
|  | Kidney stone | 814 | 18 | 18 | 2.26 | 2.21 |
|  | Cirrhosis | 814 | 0 | 18 | 0.00 | 2.21 |
|  | Hepatic cyst | 814 | 47 | 18 | 5.90 | 2.21 |
|  | COPD | 814 | 9 | 0 | 1.11 | 0.00 |
|  | CVDs | 814 | 12 | 35 | 1.54 | 4.30 |
| **Coal digger** | Overweight | 1321 | 601 | 1 | 45.53 | 0.08 |
|  | Obesity | 1321 | 285 | 1 | 21.59 | 0.08 |
|  | Hypertension | 1321 | 252 | 215 | 22.78 | 16.28 |
|  | Type 2 diabetes | 1321 | 81 | 535 | 10.31 | 40.50 |
|  | Hyperuricemia | 1321 | 352 | 3 | 26.71 | 0.23 |
|  | Hyperlipidemia | 1321 | 579 | 11 | 44.20 | 0.83 |
|  | Gallstone | 1321 | 19 | 11 | 1.45 | 0.83 |
|  | Fatty liver | 1321 | 313 | 11 | 23.89 | 0.83 |
|  | Kidney stone | 1321 | 23 | 11 | 1.76 | 0.83 |
|  | Cirrhosis | 1321 | 1 | 11 | 0.08 | 0.83 |
|  | Hepatic cyst | 1321 | 82 | 11 | 6.26 | 0.83 |
|  | COPD | 1321 | 9 | 0 | 0.68 | 0.00 |
|  | CVDs | 1321 | 20 | 31 | 1.55 | 2.35 |
| **Hydraulic support operator** | Overweight | 266 | 126 | 0 | 47.37 | 0.00 |
|  | Obesity | 266 | 38 | 0 | 14.29 | 0.00 |
|  | Hypertension | 266 | 28 | 151 | 24.35 | 56.77 |
|  | Type 2 diabetes | 266 | 12 | 132 | 8.96 | 49.62 |
|  | Hyperuricemia | 266 | 66 | 0 | 24.81 | 0.00 |
|  | Hyperlipidemia | 266 | 106 | 0 | 39.85 | 0.00 |
|  | Gallstone | 266 | 4 | 0 | 1.50 | 0.00 |
|  | Fatty liver | 266 | 45 | 0 | 16.92 | 0.00 |
|  | Kidney stone | 266 | 5 | 0 | 1.88 | 0.00 |
|  | Cirrhosis | 266 | 0 | 0 | 0.00 | 0.00 |
|  | Hepatic cyst | 266 | 18 | 0 | 6.77 | 0.00 |
|  | COPD | 266 | 8 | 0 | 3.01 | 0.00 |
|  | CVDs | 266 | 2 | 23 | 0.82 | 8.65 |
| **Belt operator** | Overweight | 1193 | 534 | 2 | 44.84 | 0.17 |
|  | Obesity | 1193 | 289 | 2 | 24.27 | 0.17 |
|  | Hypertension | 1193 | 188 | 333 | 21.86 | 27.91 |
|  | Type 2 diabetes | 1193 | 88 | 617 | 15.28 | 51.72 |
|  | Hyperuricemia | 1193 | 369 | 3 | 31.01 | 0.25 |
|  | Hyperlipidemia | 1193 | 583 | 8 | 49.20 | 0.67 |
|  | Gallstone | 1193 | 25 | 8 | 2.11 | 0.67 |
|  | Fatty liver | 1193 | 354 | 8 | 29.87 | 0.67 |
|  | Kidney stone | 1193 | 31 | 8 | 2.62 | 0.67 |
|  | Cirrhosis | 1193 | 0 | 8 | 0.00 | 0.67 |
|  | Hepatic cyst | 1193 | 59 | 8 | 4.98 | 0.67 |
|  | COPD | 1193 | 15 | 0 | 1.26 | 0.00 |
|  | CVDs | 1193 | 20 | 78 | 1.79 | 6.54 |
| **Safety inspector** | Overweight | 1326 | 624 | 0 | 47.06 | 0.00 |
|  | Obesity | 1326 | 351 | 0 | 26.47 | 0.00 |
|  | Hypertension | 1326 | 203 | 463 | 23.52 | 34.92 |
|  | Type 2 diabetes | 1326 | 112 | 642 | 16.37 | 48.42 |
|  | Hyperuricemia | 1326 | 443 | 0 | 33.41 | 0.00 |
|  | Hyperlipidemia | 1326 | 725 | 8 | 55.01 | 0.60 |
|  | Gallstone | 1326 | 29 | 9 | 2.20 | 0.68 |
|  | Fatty liver | 1326 | 513 | 9 | 38.95 | 0.68 |
|  | Kidney stone | 1326 | 34 | 9 | 2.58 | 0.68 |
|  | Cirrhosis | 1326 | 2 | 9 | 0.15 | 0.68 |
|  | Hepatic cyst | 1326 | 75 | 9 | 5.69 | 0.68 |
|  | COPD | 1326 | 13 | 0 | 0.98 | 0.00 |
|  | CVDs | 1326 | 22 | 68 | 1.75 | 5.13 |
| **Locomotive driver** | Overweight | 1658 | 720 | 2 | 43.48 | 0.12 |
|  | Obesity | 1658 | 401 | 2 | 24.21 | 0.12 |
|  | Hypertension | 1658 | 250 | 592 | 23.45 | 35.71 |
|  | Type 2 diabetes | 1658 | 132 | 787 | 15.15 | 47.47 |
|  | Hyperuricemia | 1658 | 497 | 5 | 30.07 | 0.30 |
|  | Hyperlipidemia | 1658 | 876 | 11 | 53.19 | 0.66 |
|  | Gallstone | 1658 | 42 | 12 | 2.55 | 0.72 |
|  | Fatty liver | 1658 | 517 | 12 | 31.41 | 0.72 |
|  | Kidney stone | 1658 | 51 | 12 | 3.10 | 0.72 |
|  | Cirrhosis | 1658 | 0 | 12 | 0.00 | 0.72 |
|  | Hepatic cyst | 1658 | 101 | 12 | 6.14 | 0.72 |
|  | COPD | 1658 | 16 | 0 | 0.97 | 0.00 |
|  | CVDs | 1658 | 29 | 70 | 1.83 | 4.22 |
| **Flotation worker** | Overweight | 323 | 135 | 0 | 41.80 | 0.00 |
|  | Obesity | 323 | 83 | 0 | 25.70 | 0.00 |
|  | Hypertension | 323 | 34 | 245 | 43.59 | 75.85 |
|  | Type 2 diabetes | 323 | 30 | 78 | 12.24 | 24.15 |
|  | Hyperuricemia | 323 | 95 | 2 | 29.60 | 0.62 |
|  | Hyperlipidemia | 323 | 145 | 19 | 47.70 | 5.88 |
|  | Gallstone | 323 | 10 | 19 | 3.29 | 5.88 |
|  | Fatty liver | 323 | 105 | 19 | 34.54 | 5.88 |
|  | Kidney stone | 323 | 4 | 19 | 1.32 | 5.88 |
|  | Cirrhosis | 323 | 1 | 19 | 0.33 | 5.88 |
|  | Hepatic cyst | 323 | 27 | 19 | 8.88 | 5.88 |
|  | COPD | 323 | 5 | 0 | 1.55 | 0.00 |
|  | CVDs | 323 | 4 | 11 | 1.28 | 3.41 |
| **Heavy media sorter** | Overweight | 197 | 79 | 0 | 40.10 | 0.00 |
|  | Obesity | 197 | 43 | 0 | 21.83 | 0.00 |
|  | Hypertension | 197 | 41 | 34 | 25.15 | 17.26 |
|  | Type 2 diabetes | 197 | 20 | 69 | 15.63 | 35.03 |
|  | Hyperuricemia | 197 | 51 | 1 | 26.02 | 0.51 |
|  | Hyperlipidemia | 197 | 86 | 3 | 44.33 | 1.52 |
|  | Gallstone | 197 | 7 | 2 | 3.59 | 1.02 |
|  | Fatty liver | 197 | 53 | 2 | 27.18 | 1.02 |
|  | Kidney stone | 197 | 5 | 2 | 2.56 | 1.02 |
|  | Cirrhosis | 197 | 0 | 2 | 0.00 | 1.02 |
|  | Hepatic cyst | 197 | 16 | 2 | 8.21 | 1.02 |
|  | COPD | 197 | 3 | 0 | 1.52 | 0.00 |
|  | CVDs | 197 | 3 | 26 | 1.75 | 13.20 |
| **Electrician/maintenance worker** | Overweight | 1608 | 720 | 2 | 44.83 | 0.12 |
|  | Obesity | 1608 | 347 | 2 | 21.61 | 0.12 |
|  | Hypertension | 1608 | 258 | 446 | 22.20 | 27.74 |
|  | Type 2 diabetes | 1608 | 89 | 932 | 13.17 | 57.96 |
|  | Hyperuricemia | 1608 | 522 | 3 | 32.52 | 0.19 |
|  | Hyperlipidemia | 1608 | 748 | 23 | 47.19 | 1.43 |
|  | Gallstone | 1608 | 32 | 25 | 2.02 | 1.55 |
|  | Fatty liver | 1608 | 434 | 25 | 27.42 | 1.55 |
|  | Kidney stone | 1608 | 38 | 25 | 2.40 | 1.55 |
|  | Cirrhosis | 1608 | 0 | 25 | 0.00 | 1.55 |
|  | Hepatic cyst | 1608 | 83 | 25 | 5.24 | 1.55 |
|  | COPD | 1608 | 18 | 0 | 1.12 | 0.00 |
|  | CVDs | 1608 | 18 | 75 | 1.17 | 4.66 |
| **Water pumper** | Overweight | 491 | 188 | 0 | 38.29 | 0.00 |
|  | Obesity | 491 | 121 | 0 | 24.64 | 0.00 |
|  | Hypertension | 491 | 43 | 265 | 19.03 | 53.97 |
|  | Type 2 diabetes | 491 | 31 | 276 | 14.42 | 56.21 |
|  | Hyperuricemia | 491 | 132 | 0 | 26.88 | 0.00 |
|  | Hyperlipidemia | 491 | 245 | 33 | 53.49 | 6.72 |
|  | Gallstone | 491 | 17 | 34 | 3.72 | 6.92 |
|  | Fatty liver | 491 | 137 | 34 | 29.98 | 6.92 |
|  | Kidney stone | 491 | 15 | 34 | 3.28 | 6.92 |
|  | Cirrhosis | 491 | 0 | 34 | 0.00 | 6.92 |
|  | Hepatic cyst | 491 | 20 | 34 | 4.38 | 6.92 |
|  | COPD | 491 | 3 | 0 | 0.61 | 0.00 |
|  | CVDs | 491 | 6 | 26 | 1.29 | 5.30 |
| **Others** | Overweight | 1858 | 789 | 7 | 42.63 | 0.38 |
|  | Obesity | 1858 | 417 | 7 | 22.53 | 0.38 |
|  | Hypertension | 1858 | 254 | 750 | 22.92 | 40.37 |
|  | Type 2 diabetes | 1858 | 121 | 996 | 14.04 | 53.61 |
|  | Hyperuricemia | 1858 | 531 | 15 | 28.81 | 0.81 |
|  | Hyperlipidemia | 1858 | 985 | 46 | 54.36 | 2.48 |
|  | Gallstone | 1858 | 34 | 39 | 1.87 | 2.10 |
|  | Fatty liver | 1858 | 559 | 39 | 30.73 | 2.10 |
|  | Kidney stone | 1858 | 45 | 39 | 2.47 | 2.10 |
|  | Cirrhosis | 1858 | 0 | 39 | 0.00 | 2.10 |
|  | Hepatic cyst | 1858 | 109 | 39 | 5.99 | 2.10 |
|  | COPD | 1858 | 21 | 0 | 1.13 | 0.00 |
|  | CVDs | 1858 | 27 | 68 | 1.51 | 3.66 |

^a^ NCDs, non-communicable diseases; COPD, chronic obstructive pulmonary disease; CVDs, cardiovascular diseases.
^b^ The definitions for NCDs are described in Supplementary Table S2.
**^c^** Prevalence estimates were calculated using participants with available data for the relevant outcome within each detailed occupational group as the denominator, whereas missing-data percentages were calculated using the total number of participants in the corresponding detailed occupational group as the denominator.
